# Engaging Zimbabwean men and stakeholders in the co-adaptation of peer-delivered HIV self-testing: iterative prototyping of the IMPERATIVE Trial

**DOI:** 10.64898/2026.08.10.26360082

**Published:** 2026-08-11

**Authors:** Maureen McGowan, Rufurwokuda Maswera, Louis Chisvo, Louisa Moorhouse, Freedom Dzamatira, Phyllis Mandizvidza, Blessing Tsenesa, Wilfred Otambo, Maxime Inghels, Guy Harling, Paul Mee, Till Bärnighausen, Simon Gregson, Constance Nyamukapa, Frank Tanser, Morten Skovdal

## Abstract

**Introduction:**

HIV testing and pre-exposure prophylaxis (PrEP) are efficacious HIV prevention strategies, but uptake remains low among Sub-Saharan African men. Peer-delivered approaches may improve engagement. We developed an intervention combining peer-delivered oral HIV self-testing (HIVST) with incentivized peer referral to HIV services and an SMS-based HIV risk assessment among men in eastern Zimbabwe (IMPERATIVE Trial: NCT06370923 ). We co-adapted the intervention through iterative prototyping (IP) to enhance its acceptability, feasibility, and potential effectiveness.

**Methods:**

From November 2023 to June 2024, we implemented a novel IP framework to refine and test the intervention. Four primary distributors (PDs) were trained to deliver HIVSTs to three peers and refer them to clinic services. Peers could become secondary distributors (SDs), obtain HIVSTs from community hubs and distribute them further. Qualitative data were collected alongside intervention testing to adapt the intervention over two iterations. Activities included three forum theatre workshops, one community advisory board meeting, 25 in-depth interviews, four focus group discussions, and eight observational reports involving men, implementers, stakeholders, and advisory board members. Additionally, 20 men completed baseline and one-week follow-up surveys. Quantitative data were analysed descriptively; qualitative data were analysed using thematic analysis.

**Results:**

During testing, HIVST uptake was 100% among PDs, 90% among PD-recruited peers, and 63% among SD-recruited peers. Among self-testers, 50% sought confirmatory testing and about one-quarter initiated PrEP (PDs 25%, PD-recruited peers 30%, SD-recruited peers 25%). Participants viewed the intervention positively and anticipated increased HIV testing and PrEP initiation. Four areas for refinement were identified: recruitment, information dissemination, incentives, and socio-cultural factors. Participant recommendations were adopted before randomised controlled trial testing.

**Conclusion:**

Peer-delivered HIVST with referral to HIV services shows promise for engaging Zimbabwean men. The IP framework incorporating participant recommendations enhanced intervention design and delivery within the IMPERATIVE trial. This methodology may inform future intervention development in similar settings.

**Summary Sentences:**

**What is already known on this topic**

➢ Men in Sub-Saharan Africa, including Zimbabwe, continue to be vulnerable to HIV infection and face challenges HIV testing and initiating services at clinics. A promising method to support HIV testing is through peer-delivered HIV self-testing; yet little is known about its acceptability, feasibility and potential efficacy among heterosexual men.

**What this study adds**

➢ To enhance the peer-delivered intervention, we co-adapted the model with input from men, implementers, stakeholders, and advisory board members utilizing a novel iterative prototype testing and multi-method qualitative framework.
➢ We found that peer-delivered HIV self-testing and peer referral to HIV services may be an acceptable and feasible strategy to reach Zimbabwean men vulnerable to HIV and lead to high levels of HIV testing and pre-exposure prophylaxis uptake. Our qualitative research indicated four areas for intervention refinement (recruitment, information dissemination, incentives, and socio-cultural factors) which we adapted to enhance the intervention prior to scale-up in a randomised controlled trial.

**How this study might affect research, practice or policy**

➢ Our study indicated that a peer-delivered HIV self-testing intervention may an acceptable method to reach heterosexual men in Sub-Saharan Africa and should be considered in future intervention practice. Our iterative prototyping approach was effective for co-adapting the intervention and should be considered as a research methodology, particularly in low-resource contexts aiming to rapidly implement HIV prevention interventions.

## Full Text Availability

The license terms selected by the author(s) for this preprint version do not permit archiving in PMC. The full text is available from the preprint server.

